# Automated Esophageal Cancer Staging From Free-Text Radiology Reports: Large Language Model Evaluation Study

**DOI:** 10.2196/75556

**Published:** 2025-10-17

**Authors:** Yao Yao, Xingxing Cen, Lu Gan, Jiehui Jiang, Min Wang, Yinghui Xu, Junyi Yuan

**Affiliations:** 1 Information Center, Shanghai Chest Hospital, Shanghai Jiao Tong University School of Medicine, No 241, West Huaihai Road, Shanghai, 200030, China, 86 22200000; 2 Department of Healthcare, INF Technology, Shanghai, China; 3 School of Life Sciences, Shanghai University, Shanghai, China; 4 Artificial Intelligence Innovation and Incubation Institute of Fudan University, Fudan University, Shanghai, China

**Keywords:** large language model, esophageal cancer, cancer staging, tumor–node metastasis, TNM, radiology report

## Abstract

**Background:**

Accurate staging of esophageal cancer is crucial for determining prognosis and guiding treatment strategies, but manual interpretation of radiology reports by clinicians is prone to variability and limited accuracy, resulting in reduced staging accuracy. Recent advances in large language models (LLMs) have shown promise in medical applications, but their utility in esophageal cancer staging remains underexplored.

**Objective:**

This study aims to compare the performance of 3 locally deployed LLMs (INF-72B, Qwen2.5-72B, and LLaMA3.1-70B) and clinicians in preoperative esophageal cancer staging using free-text radiology reports.

**Methods:**

This retrospective study included 200 patients from Shanghai Chest Hospital who underwent esophageal cancer surgery from May to December 2024. The dataset consisted of 1134 Chinese free-text radiology reports. The reference standard was derived from postoperative pathological staging. A total of 3 LLMs determined tumor classification (T1-T4), node classification (N0-N3), and overall staging (I-IV) using 3 prompting strategies (zero-shot, chain-of-thought, and a proposed interpretable reasoning [IR] method). The McNemar test and Pearson chi-square test were used for comparisons.

**Results:**

INF-72B+IR achieved a superior overall staging accuracy of 61.5% and an *F*_1_-score of 0.60, substantially higher than the clinicians’ accuracy of 39.5% and *F*_1_-score of 0.39 (all *P*<.001). Qwen2.5-72B+IR also demonstrated an advantage, achieving an overall staging accuracy of 46% and an *F*_1_-score of 0.51, which was better than the clinicians’ performance (*P*<.001). LLaMA3.1-70B showed no statistically significant difference in overall staging performance compared to clinicians (all *P*>0.5)

**Conclusions:**

This study demonstrates that LLMs, particularly when guided by the proposed IR strategy, can accurately and reliably perform esophageal cancer staging from free-text radiology reports. This approach not only provides high-performance predictions but also offers a transparent and verifiable reasoning process, highlighting its potential as a valuable decision-support tool to augment human expertise in complex clinical diagnostic tasks.

## Introduction

Esophageal cancer is a type of malignant tumor in the digestive system and remains one of the leading causes of cancer-related death, seriously affecting human health worldwide [1-3]. Accurate preoperative staging of esophageal cancer is essential, as it directly impacts prognosis estimation and guides treatment decisions such as surgical resection, chemotherapy, or radiotherapy [4,5]. The tumor–node metastasis classification system is currently the standard for staging according to the eighth edition of the AJCC Tumor–Node Metastasis Classification of Malignant Tumors [6].

In clinical practice, preoperative staging of esophageal cancer relies predominantly on imaging modalities, with computed tomography (CT) and positron emission tomography (PET)–CT serving as the cornerstone examinations [7]. These imaging studies generate detailed radiology reports that contain crucial information about tumor characteristics, local invasion, and metastatic spread. Clinical tumor–node metastasis (cTNM), determined from these imaging studies, is pivotal for formulating individualized treatment plans [8].

However, a major challenge is that radiology reports are usually documented in an unstructured, free-text format [9]. These narrative reports can be ambiguous, lack key details, or vary in descriptive style among radiologists. This may result in key findings being missed or misinterpreted, leading to significant discrepancies between cTNM and the gold-standard pathological tumor–node metastasis (pTNM) determined after surgery. Moreover, the complexity and variability inherent in these reports make the process of extracting structured, actionable staging information both time-consuming and prone to human error. Thus, automatic classification of esophageal cancer stage from free-text radiology reports may provide significant benefits.

Recent advances in artificial intelligence (AI), particularly large language models (LLMs), offer promising solutions for these challenges [10-14]. LLMs are capable of understanding and processing natural language, making them well-suited for extracting structured information from free-text medical documents and supporting complex reasoning tasks in medical natural language processing [15-17]. Their strengths include parsing unstructured narratives, performing multi-step reasoning, and learning from vast medical corpora, which have enabled progress in information extraction, report classification, and automated summarization in medical contexts [18,19].

Despite these advances, research on the use of LLMs for cancer staging from radiology reports has largely focused on lung cancer, owing to the relatively straightforward criteria in imaging for that disease [20-22]. In contrast, esophageal cancer staging presents unique challenges: the anatomical complexity of the esophagus, its proximity to multiple vital structures, and the subtlety of early lymph node involvement make accurate staging from radiology reports considerably more difficult. This gap in the literature highlights the need for dedicated research into LLM applications for esophageal cancer staging.

While LLMs show great potential for medical applications, they are not without limitations. The hallucination effect, where models generate plausible but incorrect information, poses risks in health care settings where accuracy is paramount [23]. Recent studies have focused on optimizing prompting strategies and proposed approaches such as few-shot learning [24] and chain-of-thought (CoT) [25] to enhance the quality of model responses. However, as black-box models, LLMs often fail to provide transparent explanations for their outputs. This lack of explainability poses a significant challenge in gaining trust for health care applications, where a transparent decision-making process is critical.

This study aims to evaluate 3 LLMs (INF-72B, Qwen2.5-72B, and LLaMA3.1-70B) for automated esophageal cancer staging from Chinese free-text radiology reports. We introduce a novel prompting strategy called interpretable reasoning (IR) designed specifically to enhance both the accuracy and transparency of LLM-based staging decisions. By comparing LLM performance with that of clinicians, we aim to provide a foundation for future research and safe clinical translation of LLMs in cancer staging.

## Methods

### Data

This retrospective study was conducted at Shanghai Chest Hospital with data collected from 617 patients who underwent esophageal cancer surgery between May and December 2024.

Exclusion criteria were applied systematically to ensure data quality and relevance to the study objectives. First, 307 patients who had received neoadjuvant therapy were excluded, as such treatment alters the assessment of tumor size and metastasis in radiology reports, and such patients are staged using the ycTNM staging system, which is not within the scope of the current dataset [26]. Second, 70 patients lacking pathological diagnosis were excluded.

**Figure 1.**
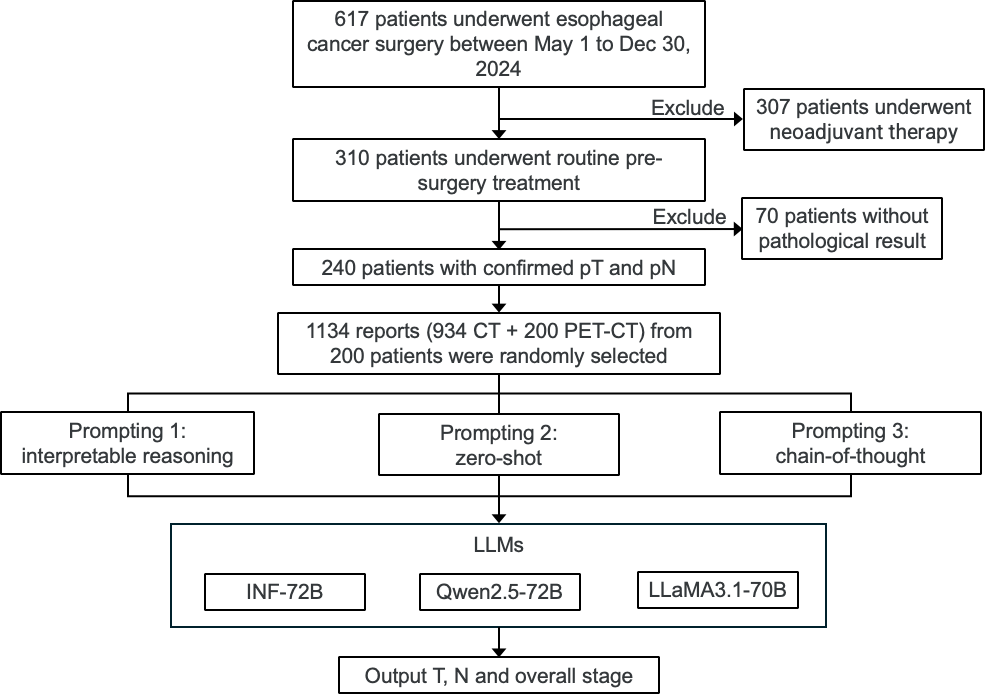
Flowchart of study design. CT: computed tomography; LLM: large language model; PET: positron emission tomography; pN: pathological node; pT: pathological tumor.

**Figure 2.**
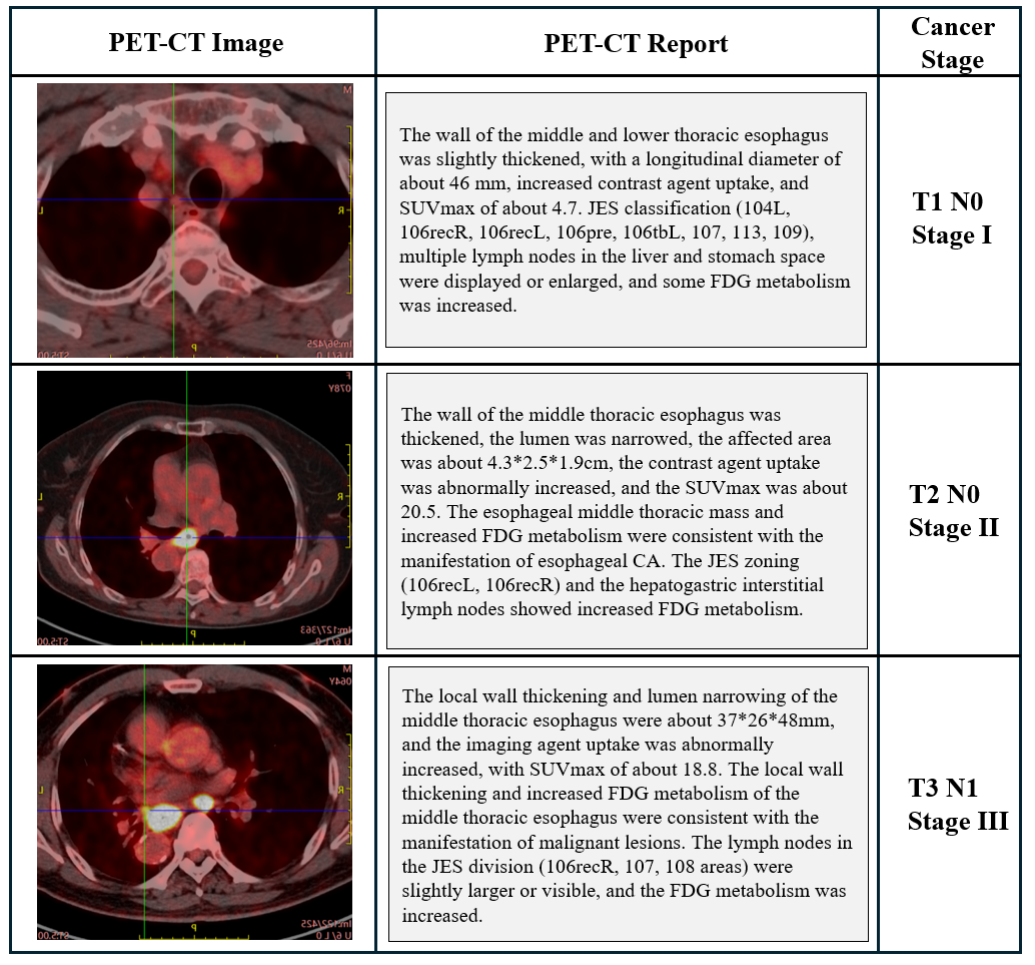
Here are 3 cases from the dataset, accompanied by the key information extracted from the free-text reports. To better illustrate the correlation between the reports and imaging, the corresponding PET-CT images are also presented. The staging of the 3 cases is T1N0 (Stage I), T2N0 (Stage II), and T3N0 (Stage III), respectively. CA: cancer; CT: computed tomography; FDG: fluorodeoxyglucose; JES: Japan Esophageal Society; PET: positron emission tomography; SUVmax: maximum standardized uptake value.

Of these, 1134 reports (934 CT and 200 PET-CT), from 200 unique patients, were randomly selected for inclusion in the final sample. Figure 1 shows the flowchart of study design. All reports were unstructured, free-text documents (Figure 2). Each case comprised all reports from the patient’s prior hospital visits, including both outpatient and inpatient records.

### Reference Standard

The reference standard for this study was the pTNM staging information obtained from each patient’s postoperative pathological report. This staging was determined by pathologists and documented within the structured pathological reporting system following surgical resection and histopathological examination.

For comparison purposes, we collected each patient’s cTNM from the admission notes completed by the attending physician prior to surgery, representing the clinician’s preoperative assessment based on available imaging reports and clinical data. After data collection, the cTNM information was reviewed by 3 attending thoracic surgeons, each with four years of clinical experience. The reviewers were not informed of the postoperative pathological staging, ensuring that their judgments relied solely on the preoperative radiology reports.

Given that our dataset consisted exclusively of surgical candidates, distant metastasis (M1) was absent, as patients with metastatic disease would not have undergone curative surgery. Therefore, our evaluation focused on tumor classification (T1-T4), node classification (N0-N3), and overall stage groups (I-IV) as determined by the combination of tumor and node categories.

### Large Language Model

The LLMs were selected for evaluation based on their availability, performance benchmarks, and number of parameters. INF-72B is a homemade LLM developed by the INF team that combines the pattern recognition capabilities of neural networks with symbolic reasoning, offering advantages for structured medical tasks [27].

Qwen2.5-72B is an open-source LLM, exhibiting strong performance in Chinese language tasks [28]. LLaMA3.1-70B is one of Meta’s foundational model series, selected for its general-purpose capabilities and widespread adoption in the research community [29]. Both are open-source models, characterized by relatively large parameter sizes and strong performance, and have been the subject of substantial research in medical text natural language processing [30,31].

All models were deployed locally within the hospital’s secure computing environment using 8 NVIDIA A30 GPUs. This local deployment was essential to ensure patient data privacy and comply with institutional data governance policies. Model inference was performed using greedy decoding (temperature=0) to maximize determinism.

### Prompting Strategy

In this study, we propose a prompting strategy called IR, in which the model is instructed not only to provide its staging decision but also to output the underlying reasoning and explicitly cite relevant excerpts from the original radiology report. This design aims to improve transparency, facilitate verification by clinicians, and reduce the risk of unsupported or hallucinatory outputs.

**Figure 3.**
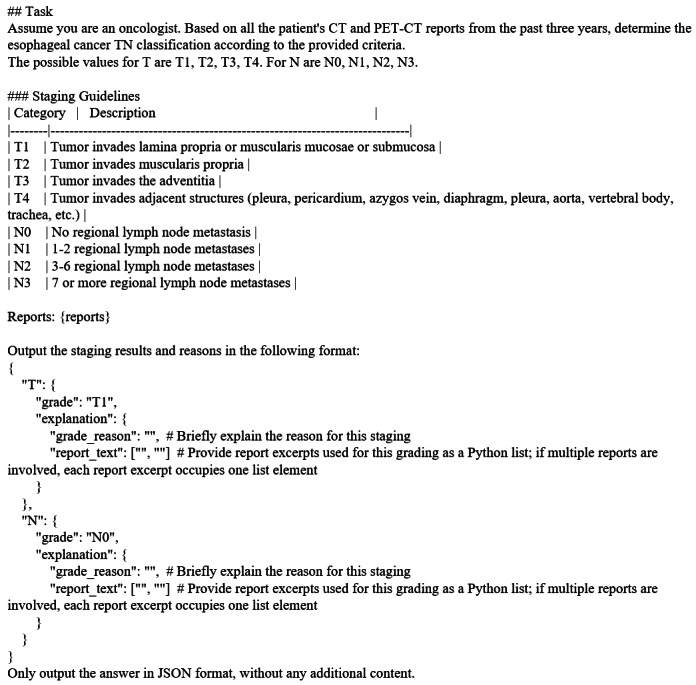
Interpretable reasoning prompt template in markdown format. CT: computed tomography; PET: positron emission tomography.

Specifically, the model’s cited excerpts from the radiology report were automatically checked against the original input text to ensure exact matches. If any cited text was not found in the original report, the output was rejected, and the model was prompted to regenerate its response. In addition, the model was instructed to perform internal reasoning before producing the final prediction and explanation, which encourages the use of evidence grounded in the report and further reduces the likelihood of hallucinations. In addition, the esophageal cancer staging rules were incorporated into the prompt in a structured format according to the AJCC Eighth Edition. The prompt template is presented in Figure 3.

A total of 2 additional prompting strategies, zero-shot (ZS) and CoT, were implemented for comparison with the proposed method. For ZS, the LLM was provided with the reports and instructions to directly return the predicted cancer stage. For CoT, the LLM was instructed to first “think step by step” to retrieve the reasoning and use it as context for predicting the cancer stage.

### Statistical Analysis

For each LLM and clinical stage, the tumor category, node category, and overall stage groups were classified as correct if matching the reference-standard assignment (pathological staging). To measure the performance of cancer staging, accuracy, precision, recall, and *F*_1_-score were used as key evaluation metrics. These metrics were calculated individually for each component (eg, T1-T4, N0-N3, and I-IV). Corresponding 95% CIs were derived using the Wilson method. Comparisons were performed using McNemar and Pearson tests. *P* values were 2-sided and considered statistically significant when less than .05.·

### Ethical Considerations

This study was approved by the Ethics Committee of Shanghai Chest Hospital (IS25024). As this was a retrospective study, the Ethics Committee of Shanghai Chest Hospital waived the requirement for participants or their legal guardians or next of kin to provide written informed consent.

## Results

### Overview

This section presents the research findings. First, we evaluated the performance of 3 LLMs in the clinical staging of esophageal cancer and compared their results with clinicians. Next, we further analyzed the outcomes in terms of clinical management categories. Finally, we compared the runtime consumption of the 3 models.

### Characteristics of Patients and Radiology Reports

A total of 200 patients (mean age 65.2, SD 7.4 years) comprising 145 males and 55 females. Within this group, a total of 1134 radiology reports (934 CT and 200 PET-CT) were collected. The distribution of pathological tumors, pathological nodes, and overall stage groups is listed in Table 1.

**Table 1.** Characteristics of patients and radiology reports.

Categories		Values (N=200)
Patient information		
Gender, n (%)		
Male		145 (72.5)
Female		55 (27.5)
Age (years), mean (SD)		65.2 (7.4)
Report quantity, n (%)		
CT^a^		934 (82.4)
PET-CT^b^		200 (17.6)
Tumor grade, n (%)		
T1		54 (27)
T2		40 (20)
T3		98 (49)
T4		8 (4)
Node grade, n (%)		
N0		94 (47)
N1		52 (26)
N2		36 (18)
N3		18 (9)
Overall stage groups, n (%)		
I		46 (23)
II		72 (36)
III		60 (30)
IV		22 (11)

a CT: computed tomography.

b PET-CT: positron emission tomography-computed tomography.

### Performance of Large Language Models and Clinicians in Esophageal Cancer Staging

Table 2 shows the accuracies of the LLMs and clinicians in esophageal cancer staging. The clinicians achieved accuracy of 44.5%, 42%, and 39.5% for tumor classification, node classification, and overall staging, respectively. Among LLMs, INF-72B demonstrated the highest overall performance. Specifically, INF-72B+IR achieved the best accuracy, significantly outperforming the clinicians (68%, 65.5%, and 61.5%, respectively; *P*<.001). Qwen2.5-72B+IR achieved its best accuracy of 46% in overall staging, better than the clinician (46% vs 39.5%, *P*<.001). Although LLaMA3.1-70B+IR achieved higher accuracy than clinicians in tumor classification (55% vs 44.5%, *P*<.001), there was no statistically significant difference between the 2 in overall staging performance (39% vs 39.5%, *P*=.99).

**Table 2.** Staging accuracies of clinicians and large language models using different prompting strategies.

	Tumor classification		Node classification		Overall stage groups	
Evaluator	Accuracy (95%CI)	*P* value^a^	Accuracy (95%CI)	*P* value^a^	Accuracy (95%CI)	*P* value^a^
Clinicians	44.5 (43.5‐45.0)	—^b^	42.0 (41.0‐43.0)	—	39.5 (38.5‐40.0)	—
INF-72B+ZS	65.0 (65.0‐66.0)	<.001	55.0 (54.0‐56.5)	<.001	53.0 (51.5‐54.0)	<.001
INF-72B+CoT	65.0 (64.5‐66.0)	<.001	57.0 (56.0‐58.0)	<.001	57.0 (56.0‐58.0)	<.001
INF-72B+IR	68.0 (67.5‐69.0)	<.001	65.5 (64.5‐66.5)	<.001	61.5 (60.5‐62.5)	<.001
Qwen2.5-72B+ZS	42.0 (41.5‐43.0)	.12	40.5 (40.0‐41.0)	.42	42.0 (41.0‐43.0)	.15
Qwen2.5-72B+CoT	42.5 (41.0‐43.0)	.23	42.0 (41.0‐43.0)	.99	45.0 (44.0‐46.5)	.008
Qwen2.5-72B+IR	46.0 (45.0‐47.0)	.76	43.5 (42.5‐44.0)	.81	46.0 (45.0‐47.5)	<.001
LLaMA3.1-70B+ZS	49.0 (48.0‐49.5)	.007	33.0 (31.5‐34.0)	<.001	38.0 (37.0‐39.0)	.79
LLaMA3.1-70B+CoT	50.0 (48.5‐51.0)	<.001	36.0 (34.0‐37.5)	<.001	37.5 (36.5‐37.5)	.25
LLaMA3.1-70B+IR	55.0 (54.0‐56.0)	<.001	35.0 (34.0‐36.0)	<.001	39.0 (38.0‐40.0)	.99

a *P* values represent comparisons with the accuracies of clinicians.

b Not available.

Table 3 shows the performance of clinicians and LLMs using different prompting for determining overall stage groups. INF-72B+IR achieved the best performance, with the *F*_1_-score of 0.62, significantly higher than clinician’s 0.43 (*P*<.001). INF-72B+ZS and INF-72B+CoT also performed better than clinicians (0.56, 0.58, *P*<.001). Qwen2.5-72B performed well under CoT and IR, significantly better than clinicians (0.50, 0.51, *P*<.001). LLaMA3.1-70B showed no statistically significant differences from clinicians (0.42, 0.43, 0.44, all *P*>0.5). Tables S1 and S2 in Multimedia Appendix 1 present the precision, recall, and *F*_1_-score of the LLMs and clinicians for determining tumor category and node category, respectively.

**Table 3.** Performance of clinicians and large language models using different prompting strategies in overall staging. Values in parentheses represent the 95% CI. The *P* values represent comparisons with the *F*_1_-scores of clinicians.

Evaluator	Precision, (95% CI)	Recall, (95% CI)	*F*_1_-score, (95% CI)	*P* value
Clinicians	0.45 (0.43-0.44)	0.40 (0.40-0.41)	0.43 (0.42-0.45)	—
INF-72B+ZS	0.57 (0.55-0.58)	0.52 (0.50-0.53)	0.56 (0.54-0.57)	<.001
INF-72B+CoT	0.59 (0.56-0.60)	0.54 (0.50-0.58)	0.58 (0.55-0.60)	<.001
INF-72B+IR	0.64 (0.61-0.65)	0.59 (0.58-0.61)	0.62 (0.60-0.64)	<.001
Qwen2.5-72B+ZS	0.46 (0.44-0.47)	0.41 (0.36-0.46)	0.44 (0.40-0.46)	.87
Qwen2.5-72B+CoT	0.51 (0.48-0.52)	0.49 (0.48-0.49)	0.50 (0.49-0.50)	<.001
Qwen2.5-72B+IR	0.52 (0.50-0.53)	0.50 (0.49-0.52)	0.51 (0.49-0.52)	<.001
LLaMA3.1-70B+ZS	0.54 (0.51-0.57)	0.36 (0.34-0.37)	0.42 (0.40-0.43)	.97
LLaMA3.1-70B+CoT	0.50 (0.49-0.51)	0.40 (0.40-0.41)	0.43 (0.41-0.45)	.99
LLaMA3.1-70B+IR	0.55 (0.51-0.57)	0.41 (0.38-0.43)	0.44 (0.42-0.45)	.89

### Stratification by Clinical Management Categories

Table 4 summarizes the performance of LLMs using IR and clinicians in terms of clinical management categories. A total of 46 reports indicated early esophageal cancer, 132 indicated locally advanced esophageal cancer, and 22 indicated advanced esophageal cancer. For reports indicating early esophageal cancer, the proportion that was overstaged was 82.6% for clinicians versus 28.3%, 60.9%, and 82.6% for INF-72B, Qwen2.5-72B, and LLaMA3.1-70B using IR. For reports indicating locally advanced cancer, the proportion that was understaged was 2.3% for clinicians versus 9.1%, 3.8%, and 0% for the 3 LLMs, and the proportion that was overstaged was 6.8% for clinicians versus 3.0%, 2.3%, and 5.3% for the 3 LLMs. For reports indicating advanced cancer, the proportion that was understaged was 90.9% for clinicians versus 63.6%, 100%, and 77.3% for the 3 LLMs.

**Table 4.** Staging by large language models and clinicians, stratified by clinical management categories.

Categories	Early esophageal cancer (Stage I; n=46)		Locally advanced esophageal cancer (Stage II and III; n=132)			Advanced esophageal cancer (Stage IV; n=22)	
Evaluator	Correct	Overstaged	Correct	Overstaged	Understaged	Correct	Understaged
Clinicians	8 (17.4)	38 (82.6)	120 (90.9)	9 (6.8)	3 (2.3)	2 (9.1)	20 (90.9)
INF-72B+IR	33 (71.7)	13 (28.3)	116 (87.9)	4 (3)	12 (9.1)	8 (36.4)	14 (63.6)
Qwen2.5-72B+IR	18 (39.1)	28 (60.9)	124 (93.9)	3 (2.3)	5 (3.8)	0 (0)	22 (100)
LLaMA3.1-70B+IR	8 (17.4)	38 (82.6)	125 (94.7)	7 (5.3)	0 (0)	5 (22.7)	17 (77.3)

### Task Completion Times

**Figure 4.**
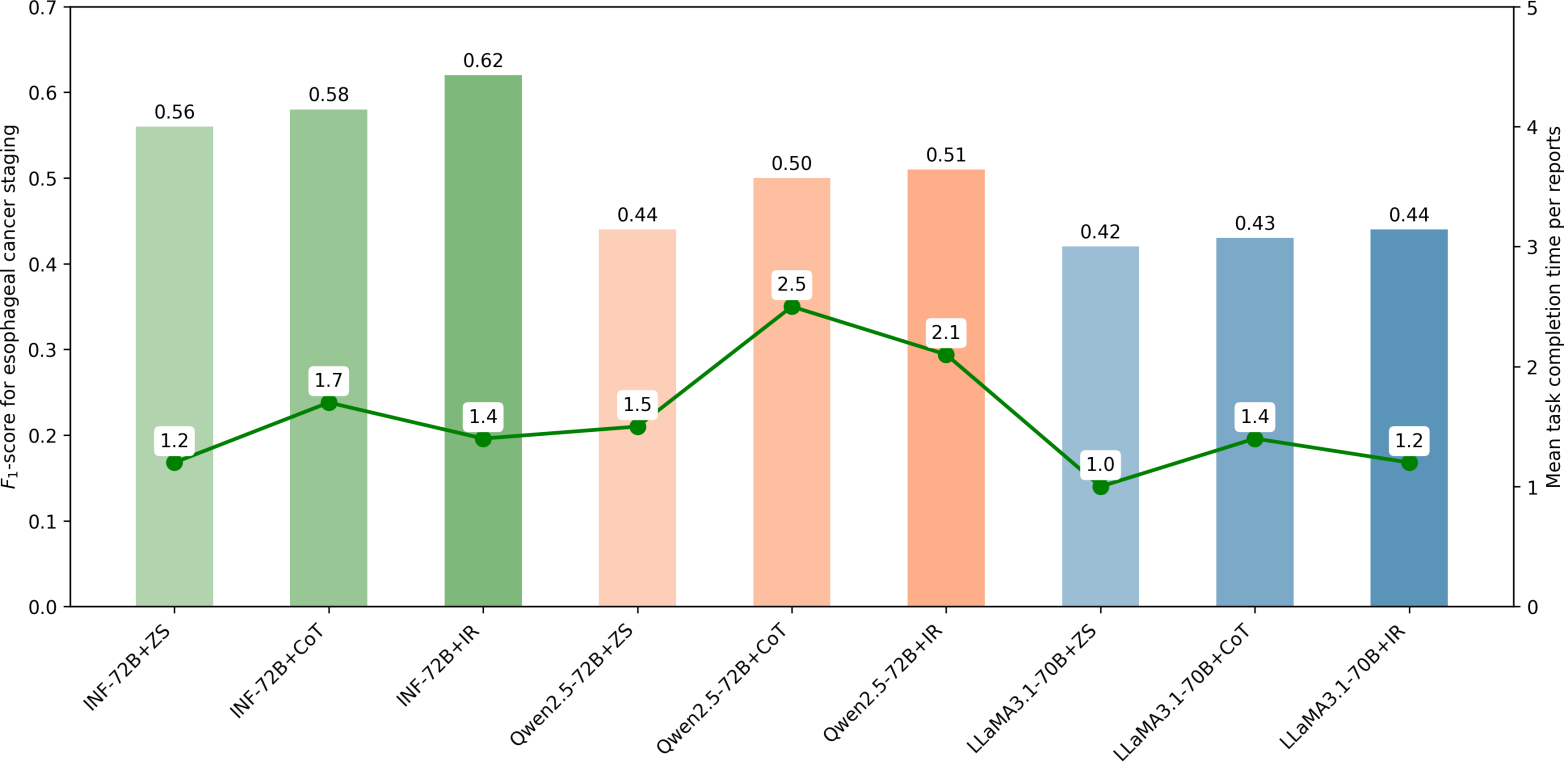
The graph shows comparisons of *F*_1_-score for esophageal cancer staging and mean task completion time per report for 3 large language models (INF-72B, Qwen2.5-72B, and LLaMA3.1-70B) with different prompting strategies (ZS, CoT, and IR). The green line and its associated values indicate mean task completion time in seconds per report for each evaluator. CoT: chain-of-thought; IR: interpretable reasoning; ZS: zero-shot.

The mean task completion times per report were 1.2 (SD 0.56), 1.7 (SD 0.58), and 1.4 (SD 0.62) seconds for INF-72B with different prompting strategies, respectively; 1.5 (SD 0.44), 2.5 (SD 0.50), and 2.1 (SD 0.51) seconds for Qwen2.5-72B, respectively; and 1 (SD 0.42), 1.4 (SD 0.43), and 1.2 (SD 0.44) seconds for LLaMA3.1-70B, respectively. Figure 4 shows the variation across LLMs in overall staging *F*_1_-score and mean task completion time per report.

### Examples of Esophageal Cancer Staging Using Large Language Model + Interpretable Reasoning

**Figure 5.**
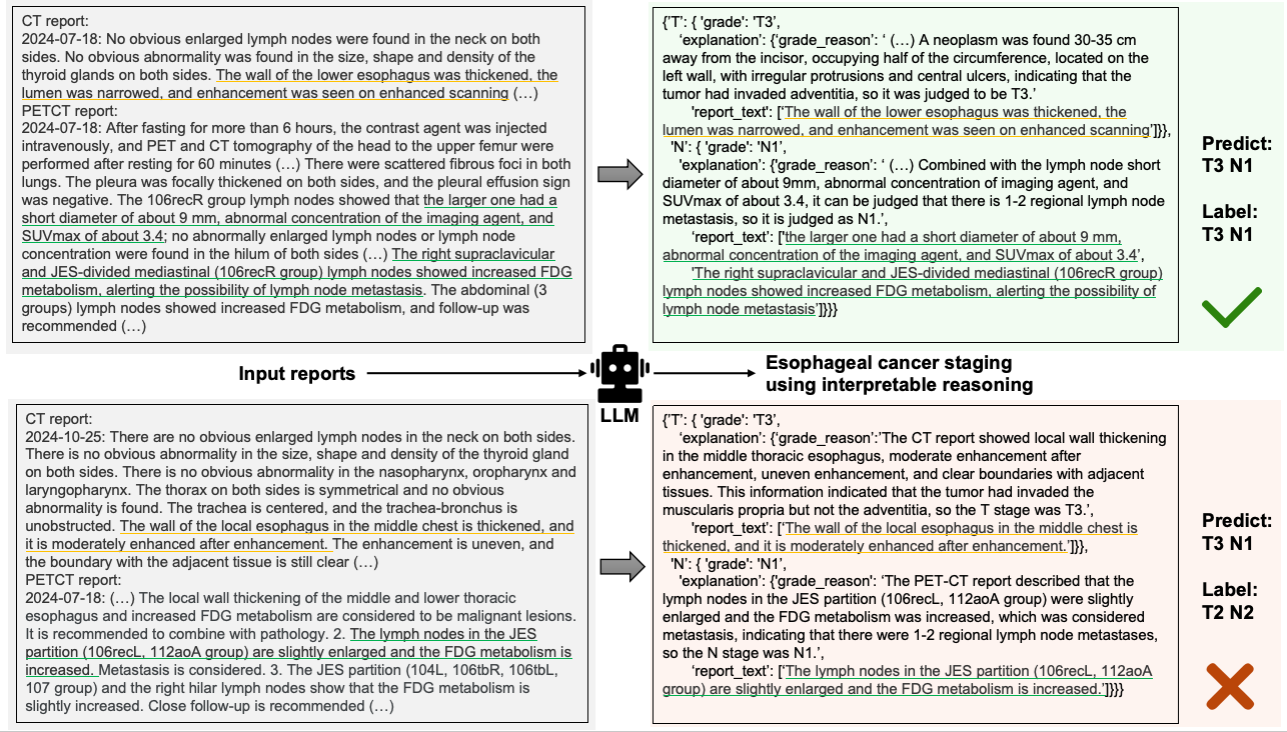
A total of 2 example cases to show how LLMs read the radiology reports and provide cancer stage using interpretable reasoning. The yellow and green underlined texts represent the classification basis of the large language model cited from the computed tomography reports and positron emission tomography-computed tomography reports, respectively. In the first example (top row), the reference standard was T3N1. The large language model extracted relevant information from the reports and gave the correct stage. In the second example (bottom row), the reference standard was T3N1. The large language model extracted incomplete information from the reports, resulting in an incorrect stage (T2N2). CT: computed tomography; FDG: fluorodeoxyglucose; JES: Japan Esophageal Society; LLM: large language model; PET: positron emission tomography.

Figure 5 shows 2 examples of esophageal cancer staging using LLM with IR, including 1 correct case and 1 incorrect case. Multimedia Appendix 1 presents the complete input and output of the model, including the prompts and the radiology reports. Appendix S2 and S3 in Multimedia Appendix 1 show another 2 error examples for tumor classifications and node classifications, including the original reports and the model’s output.

## Discussion

### Principal Findings

This study evaluated 3 LLMs for preoperative esophageal cancer staging from Chinese free-text radiology reports, comparing their performance with that of clinicians. Our proposed IR prompting strategy enabled INF-72B to achieve significantly higher accuracy and *F*_1_-scores than clinicians in tumor classification, node classification, and overall staging. Qwen2.5-72B also demonstrated improvements in overall staging under IR strategies, while LLaMA3.1-70B showed no consistent advantage over clinicians.

Recent studies have demonstrated the potential applications of LLMs in medical data analysis. In the context of radiology reports, most research has focused on using LLMs to convert free-text radiology reports into structured formats [11,18]. LLMs have been used to extract cancer stage from pathology reports, which is a relatively straightforward task due to the clearer descriptions of tumors in pathology reports [32-34]. But these approaches do not aid in formulating preoperative treatment plans for patients. Thus, this study highlights the potential of LLMs for preoperative esophageal cancer staging from radiology reports. The findings support the effective use of LLMs in assisting clinicians in real-world clinical tasks.

In this study, the IR prompting strategy played a pivotal role in enabling LLMs to clearly articulate the reasoning process behind tumor and node classification. IR guided the models to provide not only their answers but also the supporting rationale, citing key information from the original reports—such as extracting tumor status and the number of lymph nodes. This approach made the model outputs more transparent and reliable, reduced hallucinations, and highlighted the advantages of IR in complex clinical decision-making tasks such as cancer staging. Moreover, when the model produced an incorrect answer, the reasoning provided allowed for analysis of the underlying causes of the error. Such a feature is particularly valuable in the highly rigorous context of medical applications.

### Error Analysis

The predictive performance of all models for stage IV esophageal cancer was suboptimal, which may be attributable to multiple factors. First, T4 involves tumor invasion of adjacent critical structures (eg, major vessels, trachea, pericardium), where the boundaries of invasion can be difficult to delineate on imaging. Limitations in image resolution and acquisition techniques may further introduce diagnostic uncertainty, resulting in insufficient information in the radiology reports to support a T4 determination. Second, N3 is defined as distant or multiregional lymph node metastases. However, small metastatic foci or atypical lymph node morphology can be difficult to detect on conventional imaging, leading to false negatives. In addition, according to National Comprehensive Cancer Network guidelines, definitive concurrent chemoradiotherapy is recommended as the initial treatment for stage IV cases. Since this study included only patients who underwent surgery, the proportion of stage IV cases was relatively low, which limited further analysis of the contributing factors.

The models exhibited a high frequency of overstaging in cases of stage I esophageal cancer. In instances where T1 was misclassified as T2 or T3, a common pattern was LLMs often relied on report descriptions such as “localized irregular thickening of the esophageal wall with moderate, heterogeneous enhancement” as the primary basis for staging, while overlooking more critical information such as “clear demarcation from adjacent tissues” or “no mention of tumor invasion into the muscularis propria or adventitia.” Notably, INF-72B demonstrated markedly better performance in identifying stage I esophageal cancer compared with the other 2 LLMs and the clinicians, suggesting that a model’s reasoning capability can substantially influence the accuracy of esophageal cancer staging.

Errors in node classification were attributable to 2 primary causes. First, PET-CT reports sometimes lacked sufficient information to reconcile with pathological findings. Although the models correctly extracted the number of lymph node metastases documented in the PET-CT report, those counts did not always match the numbers reported in the pathology. Second, models sometimes misidentified the anatomic nodal regions when summing metastatic nodes, which led to incorrect node staging. For example, a PET-CT report might list the JES regions (104R, 104L, 106tbR, 107, 109L, 112ao, 113), whereas the model’s cited evidence mentioned only “104R, 104L, 106tbR,” omitting the other stations and thus producing a misclassification.

### Operational and Clinical Implications

INF-72B+IR demonstrated superior performance compared with clinicians in identifying both early-stage and advanced-stage esophageal cancer. This has several potential clinical benefits. First, improved recognition of early-stage disease (eg, T1N0) can facilitate timely initiation of curative-intent interventions such as endoscopic mucosal resection, endoscopic submucosal dissection, or minimally invasive esophagectomy, thereby reducing surgical morbidity and improving long-term outcomes [4]. Early and accurate identification may also prevent unnecessary chemoradiotherapy or extensive surgical procedures, reducing treatment-related complications and health care costs. Conversely, precise identification of advanced disease (eg, T4 or N3) allows for prompt initiation of multimodal treatment strategies—including concurrent chemoradiotherapy, immunotherapy, and palliative interventions—while avoiding futile surgical attempts. By reducing the risk of misclassification, the model can help ensure that patients receive stage-appropriate treatment in a timely manner.

In addition, the model processes radiology reports and generates staging predictions within seconds. This capability can improve clinical workflow efficiency by enabling a “screen-then-review” approach, in which the model prescreens cases and flags high-risk patients for expedited review by clinicians. Such an approach is particularly valuable in high-volume oncology centers, as well as in resource-limited settings. Integrating the model into multidisciplinary team discussions could further streamline decision-making, as staging information would be immediately available without requiring additional report interpretation during the meeting.

Importantly, the implementation of this model should be viewed not as a replacement for clinicians, but as an intelligent decision-support tool. By augmenting human expertise with automated, high-accuracy staging predictions, it has the potential to enhance diagnostic accuracy, reduce inter-observer variability, and optimize patient management across the entire care pathway.

### Ethical Issues in the Use of Large Language Models in Health Care

The first concern is privacy and data security. Medical information is inherently sensitive, requiring robust protection measures. In this study, all models were deployed locally within the hospital’s secure computing environment to prevent data exposure. Future implementation must ensure strong network security, compliance with regulations such as Health Insurance Portability and Accountability Act (HIPAA) and General Data Protection Regulation (GDPR), and minimal data exposure during model training and deployment. Working within these legal and ethical parameters is crucial for maintaining the credibility and trustworthiness of AI applications in health care.

In addition, the emphasis on explainable artificial intelligence methods underscores the importance of transparency to build trust among health care providers and ensure the interpretability of AI-generated outputs [35]. Building trust in AI systems requires explainable outputs and clear accountability structures. Our IR approach addresses this by providing explicit rationales for staging decisions, allowing clinicians to verify and understand model predictions. However, ongoing assessment and performance monitoring are essential for long-term accountability. Regular audits, error analysis, and feedback mechanisms must be established to maintain trustworthiness.

Another important factor is compliance with regulations. The integration of AI in health care must align with regional and international standards. This requires comprehensive documentation of model training, validation, and clinical workflow integration processes. Clear, understandable documentation enables health care providers, patients, and regulators to evaluate the technology’s ethical and legal sustainability. Our study represents an initial evaluation phase. Prospective clinical trials and regulatory approval would be necessary before deployment.

To comprehensively address these ethical considerations, ongoing assessment, open reporting, and collaboration between AI developers, health care providers, and regulators are essential. While our study demonstrates technical feasibility, the path to clinical deployment must prioritize patient rights, ensure equitable care delivery, and maintain the highest ethical standards. Only through such careful consideration can we realize the benefits of AI in health care while safeguarding the fundamental principles of medical ethics.

### Limitations

This study has several limitations that should be acknowledged. First, the most significant limitation of this study is the imbalance in the dataset. Advanced esophageal cancer cases, particularly those with T4 or N3 staging, are underrepresented because such patients often do not undergo surgical resection and thus lack postoperative pathological confirmation to serve as a reference label. This constraint prevented us from fully assessing the model’s performance in predicting advanced cancer stage. In addition, the dataset size was relatively small, which not only limits the statistical power of our findings but also precludes the possibility of performing finetuning experiments to further optimize the model.

Second, the preoperative cancer staging in this work was derived exclusively from radiology reports, with pathological pTNM staging used as the reference standard. This approach assumes that radiology report–based classification can achieve equivalence to pTNM staging. However, radiology reports may not always contain sufficient detail or clarity to support an exact stage determination. The potential discrepancy between radiological descriptions and pathological findings could have contributed to misclassification.

Another limitation has to do with the model’s generalization. The dataset was obtained from a single medical center. As a result, the linguistic style, terminology usage, and reporting conventions represented in the dataset may not fully capture the diversity of radiology reports in other hospitals, regions, or countries. This limits the immediate generalizability of the model, and further validation using multi-center, multi-national datasets is required to ensure robustness across different clinical and cultural contexts.

### Future Work

Future research should focus on addressing these limitations to improve both the accuracy and generalizability of the proposed approach. Multi-center collaborations will be essential to collect a larger and more diverse dataset, particularly including advanced-stage cases. For patients who do not undergo surgical resection, alternative labeling strategies—such as consensus annotations by multiple radiologists or incorporation of longitudinal clinical follow-up data—could be explored to approximate reliable reference standards. Increasing the dataset size would also enable supervised fine-tuning of the model, potentially yielding further performance gains over the current ZS or prompt–based approach.

Moreover, integrating structured imaging-derived features with the unstructured radiology text may help bridge the gap between radiological descriptions and pathological staging, improving classification consistency. Finally, future validation should be conducted on datasets from different institutions, regions, and languages to ensure robustness across diverse clinical settings, thereby paving the way for practical integration of the model into routine multidisciplinary workflows. In addition to retrospective validation, prospective evaluation is essential before clinical deployment. We plan to integrate the proposed model into the hospital’s information system, enabling automatic generation of stage recommendations when a patient is admitted and diagnosed with esophageal cancer. Selected clinicians will be invited to participate in a controlled pilot study to assess the model’s impact on clinical decision-making, diagnostic efficiency, and inter-observer agreement. All such testing will strictly comply with institutional review board (IRB) and ethical requirements, ensuring that patient safety and data privacy remain paramount.

### Conclusions

This study proposes an effective LLM-based approach for esophageal cancer staging. Through comparison with clinicians, INF-72B+IR demonstrated higher accuracy and *F*_1_-scores in cancer staging, highlighting its potential as an assistive tool in clinical practice. These results suggest that LLM-based methods can serve as reliable decision-support systems, complementing human expertise and potentially improving the consistency and efficiency of oncologic care. Future work will prioritize expanding the dataset, enhancing multimodal integration of imaging and text data, and validating the model across diverse clinical settings through prospective studies. Beyond esophageal cancer, the proposed framework could be extended to staging and diagnosis in other malignancies, contributing to the broader advancement of intelligent health care systems.

## Supplementary material

10.2196/75556 Multimedia Appendix 1 Additional figures, tables, and examples of model inputs and outputs.

## Abbreviations

CoT: chain-of-thought
CT: computed tomography
cTNM: clinical tumor–node metastasis
GDPR: General Data Protection Regulation
HIPAA: Health Insurance Portability and Accountability Act
IR: interpretable reasoning
LLM: large language model
PET: positron emission tomography
pTNM: pathological tumor–node metastasis
ZS: zero-shot
