# Supplementary material for: Automated Esophageal Cancer Staging From Free-Text Radiology Reports: Large Language Model Evaluation Study
Source: JMIR Med Inform. 2025 Oct 17;13:e75556. doi: 10.2196/75556 (PMC12533932; doi:10.2196/75556)
Supplement: Multimedia Appendix 1 [file medinform-v13-e75556-s001.docx]

**Figure S1.** Here are 3 cases from the dataset, accompanied by the key information extracted from the free-text reports. To better illustrate the correlation between the reports and imaging, the corresponding positron emission tomography-CT images are also presented. The staging of the 3 cases is T1N0 (Stage I), T2N0 (Stage II), and T3N0 (Stage III), respectively. PET-CT: positron emission tomography-CT.

**
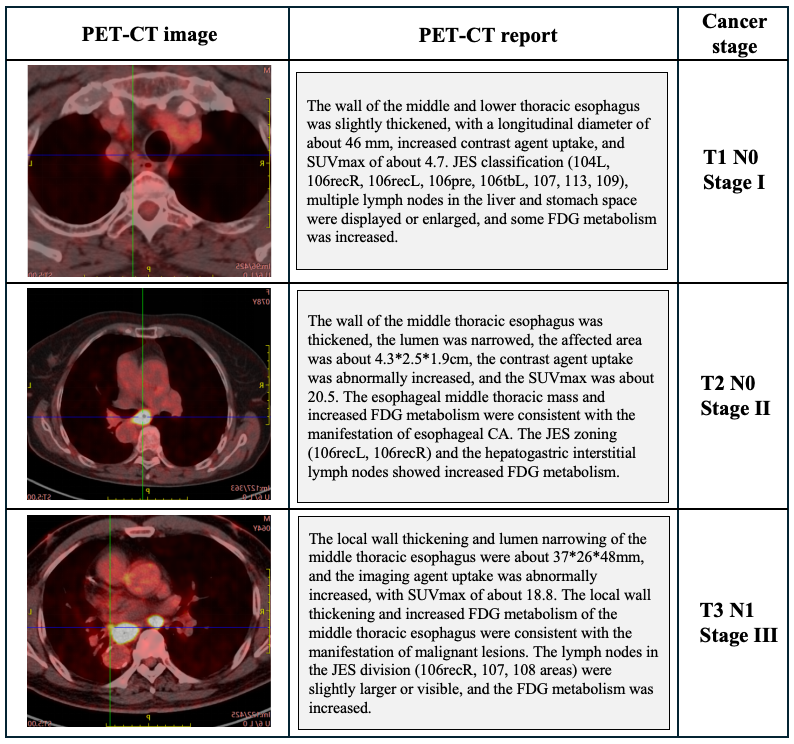
**

**Figure S2.** Interpretable reasoning prompt template in markdown format. CT: computed tomography; PET-CT: positron emission tomography-CT;

**
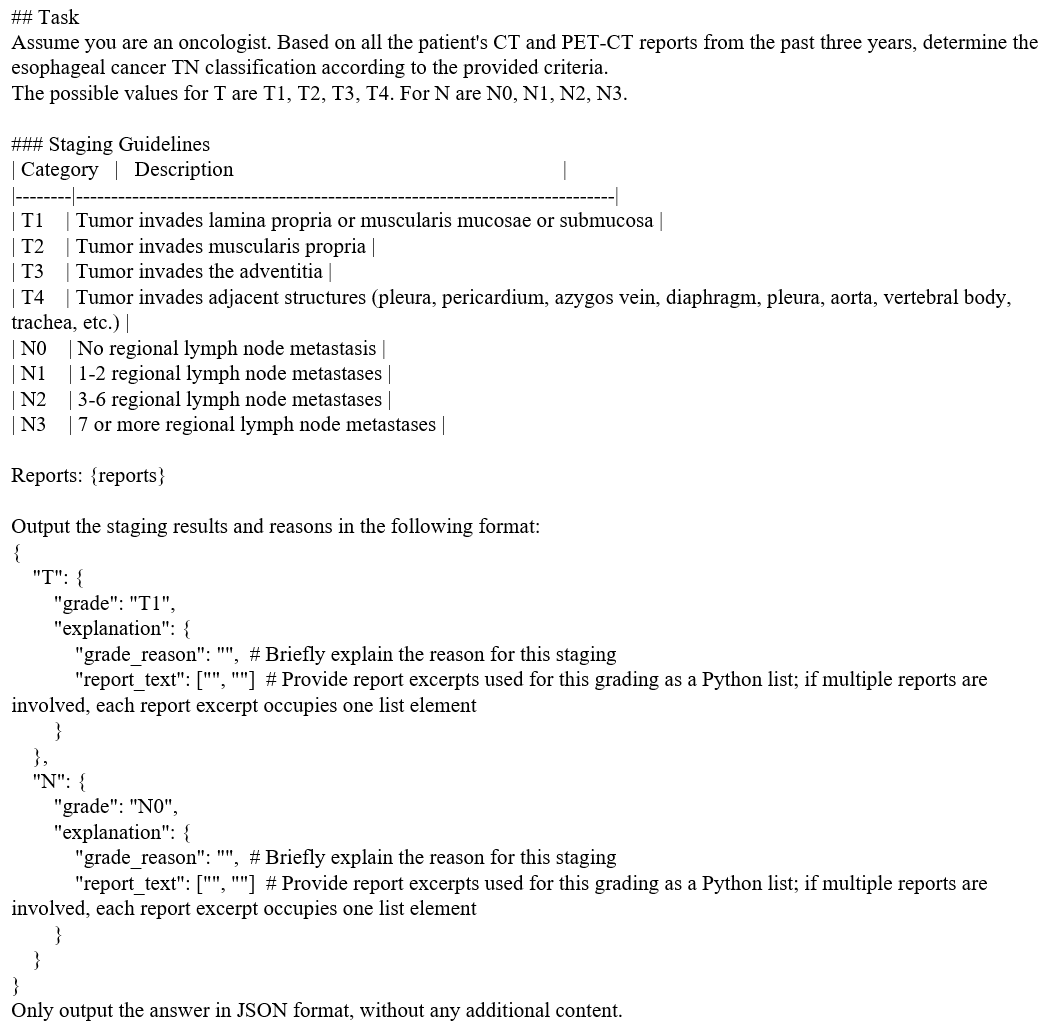
**

**Table S1.** Performance of clinical staging and LLMs using different prompting strategies in T classification.

| Evaluator | Precision | Recall | *F*_1_-score | *P* Value |
| --- | --- | --- | --- | --- |
| Clinicians | 0.57 (0.55, 0.58) | 0.43 (0.41, 0.45) | 0.45 (0.43, 0.46) | - |
| INF-72B+ZS | 0.67 (0.65, 0.68) | 0.57 (0.55, 0.59) | 0.65 (0.65, 0.66) | <.001 |
| INF-72B+CoT | 0.68 (0.65, 0.70) | 0.56 (0.55, 0.57) | 0.65 (0.65, 0.66) | <.001 |
| INF-72B+IR | 0.72 (0.70, 0.73) | 0.59 (0.58, 0.60) | 0.68 (0.67, 0.69) | <.001 |
| Qwen2.5-72B+ZS | 0.59 (0.58, 0.60) | 0.42 (0.40, 0.44) | 0.40 (0.40, 0.48) | <.001 |
| Qwen2.5-72B+CoT | 0.57 (0.56, 0.58) | 0.30 (0.28, 0.31) | 0.40 (0.49, 0.40) | <.001 |
| Qwen2.5-72B+IR | 0.64 (0.62, 0.65) | 0.40 (0.38, 0.41) | 0.43 (0.40, 0.43) | 0.65 |
| LLaMA3.1-70B+ZS | 0.60 (0.59, 0.60) | 0.50 (0.49, 0.51) | 0.54 (0.52, 0.56) | <.001 |
| LLaMA3.1-70B+CoT | 0.60 (0.59, 0.60) | 0.52 (0.49, 0.53) | 0.54 (0.50, 0.58) | <.001 |
| LLaMA3.1-70B+IR | 0.62 (0.56, 0.58) | 0.51 (0.48, 0.52) | 0.56 (0.54, 0.58) | <.001 |

Note: Values in parentheses represent the 95% CI. The *P* values represent comparisons with the *F*_1_-scores of clinicians.

**Table S2.** Performance of clinical staging and LLMs using different prompting strategies in N classification.

| Evaluator | Precision | Recall | *F*_1_-score | *P* Value |
| --- | --- | --- | --- | --- |
| Clinicians | 0.45 (0.44, 0.46) | 0.42 (0.41, 0.43) | 0.42 (0.41, 0.43) | - |
| INF-72B+ZS | 0.56 (0.54, 0.60) | 0.44 (0.38, 0.48) | 0.55 (0.49, 0.59) | <.001 |
| INF-72B+CoT | 0.61 (0.59, 0.63) | 0.48 (0.46, 0.50) | 0.60 (0.58, 0.62) | <.001 |
| INF-72B+IR | 0.66 (0.64, 0.67) | 0.52 (0.49, 0.55) | 0.65 (0.63, 0.66) | <.001 |
| Qwen2.5-72B+ZS | 0.44 (0.42, 0.45) | 0.39 (0.36, 0.40) | 0.39 (0.36, 0.40) | 0.08 |
| Qwen2.5-72B+CoT | 0.44 (0.42, 0.45) | 0.43 (0.39, 0.47) | 0.42 (0.40, 0.47) | 0.99 |
| Qwen2.5-72B+IR | 0.46 (0.44, 0.48) | 0.44 (0.40, 0.46) | 0.44 (0.42, 0.46) | 0.34 |
| LLaMA3.1-70B+ZS | 0.46 (0.45, 0.47) | 0.30 (0.29, 0.34) | 0.34 (0.32, 0.36) | <.001 |
| LLaMA3.1-70B+CoT | 0.47 (0.45, 0.48) | 0.33 (0.30, 0.35) | 0.36 (0.34, 0.35) | <.001 |
| LLaMA3.1-70B+IR | 0.46 (0.45, 0.47) | 0.33 (0.32, 0.33) | 0.35 (0.33, 0.37) | <.001 |

Note: Values in parentheses represent the 95% CI. The *P* values represent comparisons with the *F*_1_-scores of clinicians.

**Appendix S1.** Complete input and output of them model, including the Interpretable Reasoning prompt and the radiology reports

| **INPUT**  ## Task  Assume you are an oncologist. Based on all the patient's CT and PET-CT reports from the past three years, determine the esophageal cancer TN classification according to the provided criteria.  The possible values for T are T1, T2, T3, T4. For N are N0, N1, N2, N3.  ### Staging Guidelines  \|Category \| Description \|  \|--------\|-----------------------------------------------------------------------------\|  \| T1 \| Tumor invades lamina propria or muscularis mucosae or submucosa \|  \| T2 \| Tumor invades muscularis propria \|  \| T3 \| Tumor invades the adventitia \|  \| T4 \| Tumor invades adjacent structures (pleura, pericardium, azygos vein, diaphragm, pleura, aorta, vertebral body, trachea, etc.) \|  \| N0 \| No regional lymph node metastasis \|  \| N1 \| 1-2 regional lymph node metastases \|  \| N2 \| 3-6 regional lymph node metastases \|  \| N3 \| 7 or more regional lymph node metastases \|  CT Reports:  Findings:  Scattered patchy, linear, and micronodular shadows are seen in both lungs. Scattered emphysematous changes and bullae are observed in both lungs. A nodule in the anterior mediastinum measuring approximately 12 mm in its longest diameter. No obvious enlarged lymph nodes are seen in the bilateral hila or mediastinum. Localized thickening of the pleura on both sides. No pleural effusion is detected in either pleural cavity.  Impression:  Small nodule in the anterior mediastinum—recommend enhanced MRI for further evaluation. Fibrotic foci and micronodules in both lungs. Scattered emphysema and bullous changes in both lungs. Follow-up suggested.  PET-CT Reports:  Findings:  After fasting for more than 6 hours, the radiotracer was injected intravenously. After a 60-minute rest period, PET and CT scans from the head to the proximal femur were performed. PET images were attenuation-corrected and reconstructed, then displayed in multiple planes and slices combined with CT images.  The brain shows normal morphology with uniform radiotracer distribution in the cortical lobes. Subcortical nuclei are clearly visualized with symmetrical tracer uptake. Corresponding CT shows no significant widening or deepening of the cerebral sulci and gyri; no obvious hypodense lesions in the white matter; midline structures are not shifted. Ventricles are not enlarged, and basal ganglia appear symmetrical. Cerebellar imaging is normal and symmetrical bilaterally.  No abnormal radiotracer accumulation is seen in the nasopharynx. Bilateral oropharyngeal glands show symmetrical uptake. The thyroid lobes are normal in size and shape, with no abnormal uptake or enhanced lesions in the glandular parenchyma. No obvious abnormal lymph node accumulation in the neck.  Both lungs are clearly visualized, showing scattered fibrotic stripes and miliary nodules; no abnormal increase in tracer uptake. Diffuse cystic air-density shadows are seen in both lungs, partially fused into patches. Localized thickening of bilateral pleura; no pleural effusion detected. Cardiac muscle shows clear imaging. A small soft tissue density nodule approximately 12 x 9 mm in size with clear borders is observed in the anterior mediastinum; no abnormal tracer uptake noted.  The middle and lower thoracic esophageal wall is slightly thickened, involving approximately 46 mm in length, with increased tracer uptake; maximum standardized uptake value (SUVmax) is about 4.7. Multiple lymph nodes in JES stations (104L, 106recR, 106recL, 106pre, 106tbL, 107, 113, 109), and the hepatogastric space appear enlarged or visible, some with increased tracer uptake; the largest is located in station 109R, measuring approximately 13 mm in diameter, SUVmax about 7.0.  The stomach is well distended with normal gastric wall imaging. The liver has normal morphology and smooth contours with normal lobar proportions, though tracer distribution within the hepatic parenchyma is slightly uneven. No dilation of intra- or extrahepatic bile ducts. Gallbladder is normal in size and density, with no wall thickening. Porta hepatis structures are normal. The pancreas shows normal shape and tracer distribution; pancreatic duct is not dilated. The spleen is mildly visualized with uniform tracer uptake. Both kidneys show normal imaging with homogeneous parenchymal density; no dilation of the renal pelvis, calyces, or ureters. Bilateral adrenal glands appear generally normal.  Strip-like bowel shadows are visible in the abdomen, especially pronounced in the colon. No ascites. No obvious abnormal imaging in the abdominal wall soft tissues.  The urinary bladder in the pelvis shows normal tracer accumulation with no wall thickening. No abnormal lymph nodes in bilateral inguinal regions. The prostate is normal in size with generally normal tracer distribution.  Multiple spinal vertebrae show increased marginal bone density with osteophyte formation; no abnormal tracer uptake. A low-density nodular lesion is seen in the left iliac bone, with no abnormal tracer uptake. The joints of the upper and lower limbs appear generally normal. No abnormal tracer uptake is seen in the skin or soft tissues within the field of view.  Impression:  Slight thickening of the middle and lower thoracic esophageal wall with increased FDG metabolism, consistent with malignancy.  Multiple lymph nodes in JES stations (104L, 106recR, 106recL, 106pre, 106tbL, 107, 113, 109) and hepatogastric space showing enlargement or visibility, some with increased FDG uptake—metastatic involvement should be suspected.  Chronic inflammatory changes in both lungs; emphysema with bullae formation.  Localized thickening of bilateral pleura.  Small nodule in the anterior mediastinum without abnormal FDG uptake, likely benign.  Multiple degenerative changes in the spine.  Patchy low-density lesion in the left iliac bone without abnormal FDG uptake, likely benign.  Output the staging results and reasons in the following format:  {  "T": {  "grade": "T1",  "explanation": {  "grade_reason": "", # Briefly explain the reason for this staging  "report_text": ["", ""] # Provide report excerpts used for this grading as a Python list; if multiple reports are involved, each report excerpt occupies one list element  }  },  "N": {  "grade": "N0",  "explanation": {  "grade_reason": "", # Briefly explain the reason for this staging  "report_text": ["", ""] # Provide report excerpts used for this grading as a Python list; if multiple reports are involved, each report excerpt occupies one list element  }  }  }  Only output the answer in JSON format, without any additional content.  **OUTPUT**  {  "T": {  "grade": "T1",  "explanation": {  "grade_reason": "Slight thickening of the middle and lower thoracic esophageal wall, involving approximately 46 mm in length, with increased radiotracer uptake, SUVmax approximately 4.7, consistent with features of a T1-stage tumor invading the muscularis propria, muscularis mucosae, or submucosa.",  "report_text": ["Slight thickening of the middle and lower thoracic esophageal wall, involving approximately 46 mm in length, with increased radiotracer uptake, SUVmax approximately 4.7."]  }  },  "N": {  "grade": "N3",  "explanation": {  "grade_reason": "The PET-CT report describes multiple lymph nodes in JES stations 104L, 106recR, 106recL, 106pre, 106tbL, 107, 113, and 109 showing enlargement or visibility, with some exhibiting increased FDG uptake (maximum SUV ~7.0). The largest lymph node is located at station 109R, measuring approximately 13 mm in diameter.",  "report_text": ["Multiple lymph nodes in JES stations (104L, 106recR, 106recL, 106pre, 106tbL, 107, 113, 109) and hepatogastric space appear enlarged or visible, some with increased tracer uptake, "the largest is located in station 109R, measuring approximately 13 mm in diameter, SUVmax about 7.0."]  }  }  } |
| --- |

**Appendix S2.** Example free-text radiology report with discrepancy in T classification between reference standard and INF-72+IR. The original reports are in Chinese, and this appendix displays their English-translated versions. Reference standard assessment was T1. The model provided T2. Based on the model's criteria and the cited report, the model only focused on the phrases "The wall of the lower thoracic segment of the esophagus is irregularly thickened locally" and overlooked the crucial information that the "with a relatively clear boundary from adjacent tissues". This led to the misclassification of T1 as T2.

| **CT Reports**  2024-10-09  **Findings:** Both sides of the thoracic cage are symmetrical with no obvious abnormalities. The trachea is central, and the tracheobronchial tree is patent. The wall of the lower thoracic segment of the esophagus is irregularly thickened locally. It shows moderate, non-uniform enhancement after contrast administration, with a relatively clear boundary from adjacent tissues. The structure of both pulmonary hila is clear, with no obvious abnormalities in shape, size, or position. The mediastinum is central, and no abnormal enlargement of any mediastinal lymph nodes is observed. Multiple patchy shadows and calcified foci are seen in both upper lung lobes. Scattered patchy, linear opacities, and tiny nodular shadows are seen throughout both lungs. There is no significant thickening of the pleura on either side, and no significant pleural effusion is noted. Coronary artery calcification is present.  **Impression:** Occupying lesion of the lower thoracic segment of the esophagus, further specialized examination is recommended. Chronic lesions in both upper lung lobes. Scattered chronic inflammation in both lungs. Coronary artery calcification.  **Output** "T": {  "grade": "T2",  "explanation": {  "grade_reason": " The wall of the lower thoracic segment of the esophagus is irregularly thickened locally. The tumor is judged to have invaded the muscularis propria, so the T stage is T2.",  "report_text": ["The wall of the lower thoracic segment of the esophagus is irregularly thickened locally."]  }  } |
| --- |

LLM: large language model; IR: Interpretable Reasoning.

**Appendix S3.** Example free-text radiology report with discrepancy in N classification between reference standard and INF-72+IR. The original reports are in Chinese, and this appendix displays their English-translated versions. Reference standard assessment was N2. The model provided N1. Based on the model's criteria and the cited report, the model correctly extracted the information from the PET-CT report but made an error in calculating the lymph node regions, which led to an incorrect N classification.

| **PET-CT Reports**  2024-10-09  **Findings:** After fasting for over 6 hours, an intravenous imaging agent was administered. After resting quietly for 60 minutes, a PET and CT scan was performed from the head to the upper femur. The PET images were attenuation-corrected and reconstructed, and the PET and CT images were displayed in multiple planes and views.  The brain morphology is normal, with uniform distribution of the imaging agent in the cortical lobes. The subcortical nerve nuclei are clearly visible, with symmetrical uptake of the imaging agent. The simultaneous CT scan shows no significant widening or deepening of the cerebral sulci, no obvious low-density shadows in the white matter, and no midline shift. The ventricles are not enlarged, and the basal ganglia appear symmetrical. The cerebellum is normally visualized, and both cerebellar hemispheres are symmetrical.  The mucosa of both maxillary sinuses is thickened, with no abnormal uptake of the imaging agent. There is no abnormal concentration of the imaging agent in the nasopharynx, and the glands on both sides of the oropharynx are symmetrically visualized. The thyroid lobes are not enlarged and are normal in shape, with no abnormal enhancement of the imaging agent uptake in the glandular parenchyma. Lymph nodes are visible in the cervical Ib and II regions, with slightly increased uptake of the imaging agent, with a maximum standardized uptake value (SUVmax) of approximately 3.9. No obvious abnormal lymph node concentration is seen in the right supraclavicular region.  The wall of the mid-thoracic esophagus is thickened, the lumen is stenosed, and the uptake of the imaging agent is abnormally high, with an SUVmax of approximately 7.8. Lymph nodes in the JES regions (104L, 106recL, 106tbL, 106tbR, 107, 108), the hepatogastric space, and the retroperitoneum are enlarged, with the largest short axis measuring approximately 1.8 cm. The uptake of the imaging agent is abnormally high, with an SUVmax of approximately 9.9. Both lungs are clearly visualized, with normal lung markings. A tiny nodular shadow is seen in the right middle lobe, with no abnormal uptake of the imaging agent. Scattered patchy shadows are seen in both lungs. The pleura is locally thickened on both sides, and there is a negative pleural effusion sign. Lymph nodes in both pulmonary hila are visible, with slightly increased uptake of the imaging agent, with an SUVmax of approximately 3.7. The myocardium is clearly visualized.  In the abdomen, the stomach is well-filled, and the gastric wall is normally visualized. The liver is normal in shape and has a smooth contour, with a normal lobar proportion. The density of the liver parenchyma is decreased, with an average CT value of approximately 25 Hu. A calcified shadow is seen in the right liver lobe, with no abnormal uptake of the imaging agent. The intrahepatic and extrahepatic bile ducts are not dilated. The gallbladder is of normal size and uniform density, with no wall thickening. The porta hepatis structure is normal. The pancreas shows good morphology and imaging agent distribution, and the pancreatic duct is not dilated. The spleen shows slight visualization with uniform distribution of the imaging agent. Both kidneys are well-visualized with uniform parenchymal density. A cystic low-density shadow is seen in the right kidney, with a diameter of approximately 4.7 cm, and no abnormal uptake of the imaging agent. The renal pelvis, calyces, and ureters are not dilated. The adrenal glands on both sides appear generally normal. A linear intestinal shadow is visible in the abdomen, with particularly prominent visualization of the colon. The ascites sign is negative. No obvious abnormal images of the abdominal wall soft tissues are seen.  In the pelvis, the bladder shows normal concentration of the imaging agent, and the bladder wall is not thickened. No abnormal lymph nodes are seen in both inguinal regions. The prostate is of normal size with generally normal distribution of the imaging agent. A shadow with increased uptake of the imaging agent is seen locally in the rectosigmoid canal, with an SUVmax of approximately 9.4.  There is osteosclerosis on the anterior edge of the spine. No abnormal concentration of the imaging agent is seen in the axial skeleton within the field of view. The upper and lower limb joints are generally normal. No abnormal uptake of the imaging agent is seen in the skin and soft tissues within the field of view.  **Impression:** Status post-esophageal cancer treatment. The thickened wall of the mid-thoracic esophagus shows increased FDG metabolism, suggesting persistent hypermetabolic activity of the tumor after treatment. Enlarged lymph nodes in the JES regions (104L, 106recL, 108), hepatogastric space, and retroperitoneum, with increased FDG metabolism, suggesting possible lymph node metastasis. Lymph nodes in the JES regions (106tbL, 106tbR, 107) and both pulmonary hila are visible with slightly increased FDG metabolism; follow-up is recommended. Tiny nodule in the right middle lung lobe, with no abnormal FDG metabolism, suggesting a possible inflammatory nodule. Chronic inflammation of both maxillary sinuses. Lymph nodes are visible in the cervical Ib and II regions with increased FDG metabolism, suggesting possible inflammatory lymph nodes. Fatty liver. Calcified foci in the right liver lobe. Right renal cyst. Increased local FDG metabolism in the rectosigmoid canal, commonly seen in hemorrhoids. Degenerative changes of the spine.  **Output** "N": {  "grade": "N1",  "explanation": {  "grade_reason": "The PET-CT report identified enlarged lymph nodes with increased FDG metabolism in the JES partition (104L, 106recL, and 108), the hepato-gastric ligament, and the retroperitoneum. Given the definition of N staging for esophageal cancer, these findings are suspicious for lymph node metastasis and classify as N1.",  "report_text": ["Enlarged lymph nodes in the JES regions (104L, 106recL, 108), hepatogastric space, and retroperitoneum, with increased FDG metabolism, suggesting possible lymph node metastasis."]  }  } |
| --- |
| LLM: large language model; IR: Interpretable Reasoning. |
